# Improved search heuristics find 20 000 new alignments between human and mouse genomes

**DOI:** 10.1093/nar/gku104

**Published:** 2014-01-31

**Authors:** Martin C. Frith, Laurent Noé

**Affiliations:** ^1^Computational Biology Research Center, AIST, 2-4-7 Aomi, Koto-ku, Tokyo 135-0064, Japan and ^2^LIFL (UMR 8022)/INRIA Lille Nord-Europe, bat M3 Ext., Université Lille1 59655 Villeneuve d’Ascq Cedex, France

## Abstract

Sequence similarity search is a fundamental way of analyzing nucleotide sequences. Despite decades of research, this is not a solved problem because there exist many similarities that are not found by current methods. Search methods are typically based on a seed-and-extend approach, which has many variants (e.g. spaced seeds, transition seeds), and it remains unclear how to optimize this approach. This study designs and tests seeding methods for inter-mammal and inter-insect genome comparison. By considering substitution patterns of real genomes, we design sets of multiple complementary transition seeds, which have better performance (sensitivity per run time) than previous seeding strategies. Often the best seed patterns have more transition positions than those used previously. We also point out that recent computer memory sizes (e.g. 60 GB) make it feasible to use multiple (e.g. eight) seeds for whole mammal genomes. Interestingly, the most sensitive settings achieve diminishing returns for human–dog and *melanogaster–pseudoobscura* comparisons, but not for human–mouse, which suggests that we still miss many human–mouse alignments. Our optimized heuristics find ∼20 000 new human–mouse alignments that are missing from the standard UCSC alignments. We tabulate seed patterns and parameters that work well so they can be used in future research.

## INTRODUCTION

### Genome comparison is not solved

**Figure 1.**
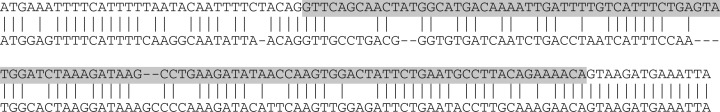
Example of a human–mouse alignment missing in the UCSC genome database. The upper sequence is from human chromosome 12, and the lower from mouse chromosome 10. The shading indicates a protein-coding exon of TSPAN19.

Genome alignments have been used for many years to study DNA evolution, and identify functional elements, positive selection, etc. So it may come as a surprise that genome alignment is not a solved problem. For example, there are thousands of human–mouse alignments that are missing from the standard UCSC alignments, one of which is shown in Figure 1. This alignment is highly significant (score = 5852, E-value = 5 × 10^−^^6^), and does not contain repetitive sequence, so is likely a true homology. Moreover, it is colinear with other alignments and is apparently a reciprocal best hit between the genomes, so is likely a one-to-one orthology. The human sequence contains a protein-coding exon of tetraspanin 19 (TSPAN19), and the alignment reveals its evolutionary fate in mouse: the frameshifting insertions/deletions suggest it has decayed into a pseudogene. Thus, each alignment tells an evolutionary story, and we would like to find them all.

### Aim of this study

The aim is to optimize search for distantly related nucleotide sequences, between large data sets (e.g. genomes). This is useful not only for genome comparison. Another application is analyzing DNA reads from a species without a reference genome, by aligning them to a genome of a different species. Yet another is alignment of metagenomic DNA reads to a set of microbial genomes. On the other hand, our results will not be relevant for finding close similarities, such as aligning human DNA reads to a human genome. We also do not focus specifically on protein-coding DNA, which other complementary studies have done (1,2).

We should also mention that there is more to genome alignment than similarity search: in particular, removal of paralogs. This study just optimizes the similarity search component.

### Scores and seeds

The standard approach to sequence similarity search has two steps:

1. Define an alignment scoring scheme.
2. Search for alignments with optimal scores.

Both are important, but this study considers only step 2. There are dynamic programming algorithms that guarantee to find alignments with optimal scores, but unfortunately they are too slow for large genomes, and so heuristic algorithms are used instead. The standard heuristic approach is ‘seed-and-extend’: this first finds ‘seeds’ (i.e. short matches that can be found quickly), and then looks for high-scoring alignments around each seed (3,4).

### Spaced seeds

The simplest kind of seed is an exact match of a given length, e.g. 11 bases. A ‘spaced seed’, on the other hand, is a match within which certain positions are allowed to mismatch. As an example, here is a spaced seed pattern of length 8 and ‘weight’ 6: 11101011. This means that we seek matches of length 8, but the fourth and sixth positions are allowed to mismatch.

It is not obvious that spaced seeds have any advantage over same-weight unspaced seeds, but it turns out that they can have higher sensitivity (1,5–7). The reason is subtle, but roughly speaking it is because overlapping seed hits are more independent.

### Transition-constrained seeds

Transitions (a
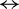
g or c
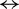
t) are often more common than transversions (all other substitutions). This motivates ‘transition-constrained seeds’ (8), which are represented by patterns like this: 11T0TT010T. Positions with T tolerate transitions but not transversions.

### Adaptive seeds

‘Adaptive seeds’ do not have a fixed length: instead, they have a rareness threshold (9). Specifically, starting from each position in the ‘query’ sequence, they are minimum-length matches that occur at most *m* times in the ‘reference’ sequence(s).

Adaptive seeds are advantageous because genomes are rife with nonuniform composition. They avoid getting a huge number of seeds in repetitive sequence, and they adapt to the information content (i.e. rareness) of the sequence.

Adaptive seeds can be combined with spaced and transition-constrained seeds, as follows. First, we (conceptually) extend the seed pattern to infinite length by cyclic repetition (e.g. 110T → 110T110T110T …). Then, we find minimum-length matches that occur at most *m* times, using prefixes of the pattern.

### Sparse seeds

‘Sparse seeds’ are a simple idea: instead of looking for seed hits starting at every position, we look for hits starting at (e.g.) every second position, or every third position (in either the query or the reference).

Sparse seeding affects speed, sensitivity and memory usage. Aligners typically use a memory-consuming index of the reference: sparse seeding in the reference reduces this memory usage. With fixed-length seeds, sparse seeding in either query or reference reduces the number of seed hits, decreasing both sensitivity and run time. With adaptive seeds, sparse seeding in the query similarly reduces the number of seed hits, but sparse seeding in the reference does not necessarily do so, because it makes seeds rarer. In all cases, it is not obvious whether the sensitivity will decrease for a given run time.

Sparse seeds have long been used to reduce memory consumption and/or run time (10,11), but no one seems to have investigated whether they increase sensitivity for a given run time. This seems conceivable because, like spaced seeds, overlapping hits are more independent of each other.

### Extension and score drop

For each seed, aligners such as BLAST and LAST first extend a gapless alignment, and only if the alignment score achieves a threshold *d* do they proceed to the much slower gapped alignment (4,9,12). The gapless extension terminates when the score drops more than *y* below its maximum. The value of *y* is potentially important: lower values make the algorithm faster but less sensitive.

### Seed design

Spaced and transition-constrained seeds come with the problem of designing an effective seed pattern. The standard approach is to specify an alignment ‘model’, and then design a pattern with high sensitivity for that model. An example of a model is gapless alignments of length 64 where each position is a match with probability 0.7, a transition with probability 0.15 or a transversion with probability 0.15. This approach has several limitations:

- For transition-constrained (and/or multiple) seeds, it is often infeasible to find in reasonable time a guaranteed optimum pattern for a given model (13). Hence we resort to heuristic optimization.
- It is unclear how relevant such a model is to real alignments (particularly the length).
- All existing design methods are for fixed-length seeds, whereas we wish to use adaptive seeds.
- Good patterns for sparse seeds are likely different from good patterns for dense seeds, but this has not been investigated before. Fortunately, the design software Iedera (14) has options for sparse seeds.

Because of these issues, after designing seeds, we perform brute-force tests of their effectiveness with real sequences.

## MATERIALS AND METHODS

### Seed design

We designed 48 single transition seed patterns (Supplementary Tables S1, S5, S6), using Iedera version 1.05 with these options:

-A 3 -B 3 -b 0.5,0.25,0.25 -r 100000 -k
-BSymbols’0T1’
-f $f -c $c -l $l -w $w,$w -s $w,$w2

-A and -B give the size of the alignment alphabet and the seed alphabet, respectively. Combined with -BSymbols’0T1’, it means that seed symbol 0 (called ‘don’t care’) detects any alignment letter, whereas T accepts only transition and match letters, and 1 only accepts match letters.

-b and -f set the background and foreground probabilities of the alignment letters (transversion, transition, match). -r is the number of seed random trials, which are optimized by a hill-climbing heuritic (-k). Seeds are designed with fixed weight (number of 1 s plus half the number of Ts), and span (-s) ranging from $w to $w2 = $w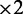.

Based on real data (see below), we set $f to either 0.12,0.18,0.7 or 0.15,0.15,0.7. $c sets the sparsity: we used 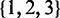. $l sets the length of gapless alignment for which the pattern is optimized: we used 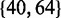. $w sets the weight: we used 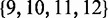.

We also made 48 sets of multiple codesigned seed patterns (Supplementary Tables S2, S3, S4), using the same options as above, except that we omitted -c $c, and added -n $n. $n sets the number of patterns: we used 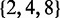.

### Genome data

We used the following genomes from UCSC (with repetitive regions indicated by lowercase): human (hg19), mouse (mm10), dog (canFam3), chicken (galGal3), *Drosophila melanogaster* (dm3) and *Drosophila pseudoobscura* (dp4) (15).

### Test procedure

Our test procedure is to take 10 000 random 1 kb chunks from one genome, and align them to another genome. These alignments are repeated using many different algorithm parameters, but a fixed scoring scheme. For each chunk, we record the highest alignment score ever seen. Then, for each alignment procedure, we count the number of chunks for which it failed to find the highest score.

Because these genomes contain large runs of NNN … , we required the random chunks to have no non-ACGT uppercase letters. Then, before aligning, we replaced each lowercase letter (in both the chunks and the target genome) with N.

The test data sets, and detailed results, are available at: http://last.cbrc.jp/dna-seed-test/.

### Alignment scoring schemes

To check the generality of our results, we used two scoring schemes.

- LAST’s default scheme: match/mismatch = +1/−1, gap exist/extend = −7/−1. In this case we used minimum alignment score = 35. An N aligned to anything gets a score of −1.
- LASTZ’s default scheme: HoxD70 matrix (16), gap exist/extend = −400/−30. In this case we used minimum alignment score = 4000. An N aligned to anything gets a score of −100.

### E-values

We calculated E-values of alignment scores (Table 1) using ALP 1.91 (17). The E-value of score *S* is the expected number of alignments with score 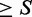 between two random sequences. Here, the length and base composition of one random sequence equals all ACGT letters (both upper- and lowercase) in one whole genome. We multiplied the E-values by 2, to reflect comparison of both DNA strands.

**Table 1.** Alignment E-values

Genomes	Scoring	Score	E-value
Human/dog	LASTZ	4000	150
Human/mouse	LASTZ	4000	120
dm3/dp4	LASTZ	4000	0.24
Human/dog	LAST	35	400
Human/mouse	LAST	35	370
dm3/dp4	LAST	35	0.66

It is not clear what E-value threshold should be used for genome comparison. An E-value of (e.g.) 400 may seem high, but genome comparison typically produces 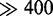 alignments, which means that only a small fraction of them are expected to be spurious. The UCSC genome alignments often have even higher E-values (18). In any case, the thresholds in our tests are not wildly unreasonable.

### LAST alignments

Most of the alignments were performed with LAST version 320. We tried all combinations of these LAST parameters:

- *m* (rareness threshold) = 10, 100, 1000. This varies sensitivity and run time because larger values of *m* get shorter and more frequent seeds, which is more sensitive but slower.
- *y* (gapless score drop) = ‘high’ or ‘low’ (see the figures).
- One hundred thirteen seed patterns:

  –Ninety-six patterns designed with Iedera.
  –Nine pairs of patterns suggested in the YASS README file (Supplementary Table S7).
  –Six single patterns from previous studies (Supplementary Table S8).
  –The trivial exact-match pattern: 111 …
  –The all-transition pattern: TTT …

Some of the Iedera patterns are designed for sparse seeding: in these cases, we used either sparse genome indexing (*w*) or sparse seeding in the query (*k*).

Because LAST cyclically extends the seed patterns, we first trimmed each pattern to its shortest prefix that recovers the original pattern on cyclic extension (Supplementary Tables S9–S16).

### LASTZ alignments

We also performed alignments with LASTZ version 1.03.42, compiled with allowBackToBackGaps=ON, which permits adjacent insertions and deletions (19). First we tried these options:

lastz_32 $genome[multiple] $queries
- -gappedthresh=4000 - -ydrop=3999
- -format=maf-

Here, ydrop is the maximum score drop for gapped extension: we set it to LAST’s default value.

Then we tried varying the *d* parameter, by adding –hspthresh=$d: we used 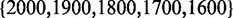.

Finally, we tried using some Iedera-designed seeds, by adding –seed=$seed –notransition.

### Whole human–mouse genome comparison

We compared the lowercase-repeat-masked genomes. From human, we first discarded alternate haplotypes, and hard-masked (replaced with N) the pseudoautosomal regions of the Y chromosome (because they are identical copies of the X chromosome). We additionally lowercase-masked simple/tandem repeats with tantan v13, to reliably avoid nonhomologous alignments (20). We then found alignments using LAST with the LASTZ scoring scheme, *y* = 962 (the default), a set of eight seeds (the second-last in Supplementary Table S12), and *m* = 100. Finally, we kept alignments with score 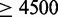 (E-value 1.6), which are likely almost all true homologs, though not necessarily orthologs.

## RESULTS

### Real transition and transversion rates

Effective seed patterns depend on the rates of transitions and transversions. We therefore counted transitions and transversions in a selection of ‘net’ genome alignments from UCSC (Table 2) (15). We should bear in mind that these alignments may not be perfect and unbiased.

**Table 2.** Transition and transversion rates for some UCSC ‘net’ genome alignments

Genomes	Identities (%)	Transitions (%)	Transversions (%)
Human/dog	76	15	9
Human/mouse	69	18	13
Human/chicken	67	17	16
*Melanogaster*/*pseudoobscura*	71	14	15

All cases show excess transitions:transversions, compared with the unbiased ratio of 1:2 (because there are twice as many possible transversions as transitions). Mammals have a greater excess than *Drosophila*, presumably because they have more methylcytosine, which mutates rapidly to thymine (21). Less-similar genomes have a lower excess of transitions: this is as expected because the transitions cannot keep increasing linearly but instead tend to an asymptote.

### Seed design

Based on the results above, we used Iedera to design transition-constrained seeds for an alignment model with identities:transitions:transversions = either 70:18:12 or 70:15:15 (Supplementary Tables S1–S6).

Interestingly, the resulting seed patterns have more Ts than those used previously (22,23). It seems that seeds were previously designed with a fixed, low number of Ts, perhaps because that simplifies their design.

### Tests of alignment heuristics

**Figure 2.**
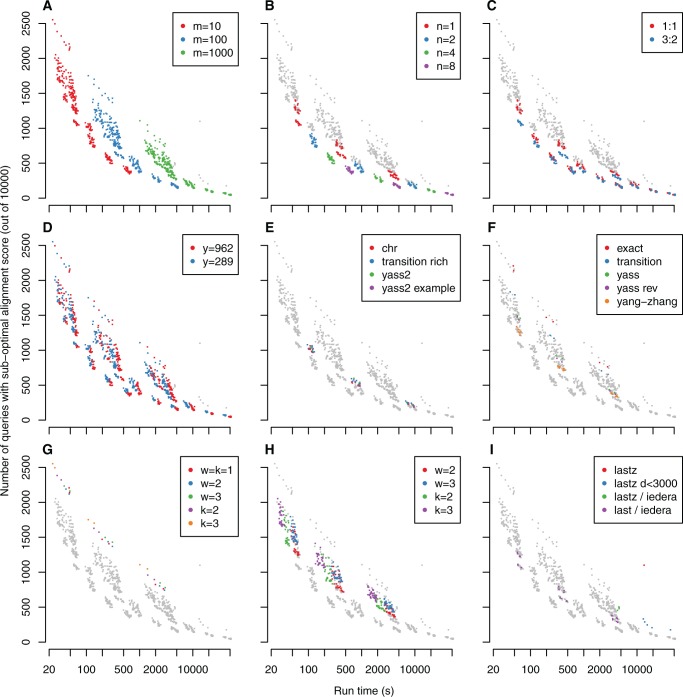
Sensitivity versus speed for aligning human queries to the dog genome with the LASTZ scoring scheme. Each point represents one alignment method. Each panel shows the same points, but highlights a different selection of them, according to: rareness threshold m (**A**), number of seeds n (**B**), transition: transversion ratio (**C**), extension threshold y (**D**), predefined seeds (**E** and **F**), sparse seeding (**G** and **H**), and software implementation choices (**I**) (see text). Points that are not highlighted are gray. Each point’s X coordinate indicates the time to align all 10 000 queries, and the Y coordinate indicates the error rate, i.e. the number of queries with suboptimal alignment score. Better performance is toward the lower-left.

Our first test was to align 10 000 random 1-kb chunks of the human genome against the dog genome. The results are shown in Figure 2, where each point is one alignment procedure, and the nine panels are identical, apart from the color scheme, which picks out various algorithm parameters.

Firstly, we examine the effect of the rareness threshold. As expected, higher values make the alignment more sensitive but slower (Figure 2A).

Secondly, we focus on the nonsparse Iedera seeds. As expected, using more codesigned seed patterns makes the alignment more sensitive but slower (Figure 2B). The interesting point, though, is that using more seeds beats increasing the rareness threshold. For example, using four seeds with 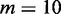 is both faster and more sensitive than one seed with 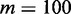. The downside is that more seeds require more memory (Table 3).

**Table 3.** Memory usage for alignment to the dog genome

Seeds	Memory (GB)
1	8
2	14
4	25
8	48

Thirdly, we compare seed patterns designed for different transition:transversion ratios. The 3:2 seeds perform better than the 1:1 seeds (Figure 2C), which is not surprising, as the actual ratio is ∼3:2 (Table 2). However, this difference decreases when we use more patterns or less-rare seeds: we do not know why.

Next we examine the effect of the gapless score drop limit (*y*). As expected, the higher value makes the alignment slower and more sensitive (Figure 2D). The difference is not great, however, and there seems little reason to favor either of the tested values.

Subsequently, we check the performance of previously suggested seed patterns. Surprisingly, we could find no previous publication that describes a set of codesigned transition seeds. However, the YASS documentation provides several pairs of codesigned seeds (Supplementary Table S7): these perform slightly worse than our Iedera 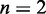 patterns (Figure 2E), presumably because they have too few Ts.

The previous single-seed patterns also perform worse than our Iedera 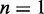 patterns (Figure 2F). Exact-match seeds are significantly worse than anything else, which is not surprising. All-transition seeds are slightly better, but still not competitive. The patterns suggested by Yang and Zhang perform well, presumably because they have more Ts than other previously suggested patterns, but our Iedera 3:2 patterns are better.

Next, we examine the effect of sparse seeding. With exact-match seeds, the sensitivity per run time becomes neither better nor worse overall (Figure 2G). To be competitive, we need to use transition seeds designed for sparsity. Sparse transition seeds, however, perform significantly worse than nonsparse seeds (Figure 2H).

### Comparison with LASTZ

A previous study found that the seeding strategy of LASTZ (and its predecessor BLASTZ) is especially effective (22). LASTZ uses this fixed-length spaced seed: 1110100110010101111, and it allows any one match position to be a transition instead. Because LASTZ is also the engine behind the UCSC genome alignments, we ran it through our test.

With default algorithmic parameters, LASTZ performed poorly, with high run time but low sensitivity (Figure 2I). However, its performance was greatly improved by simply reducing *d* (the score threshold for the gapless alignment phase) from 3000 to 2000. Still, it is not as good as LAST with multiple seeds: for example, using eight seed patterns with *m* = 100, we can get better sensitivity with less than half the run time.

Because *d* has such a large effect, we should describe its setting for LAST. By default, LAST uses 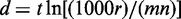, where *m* is the rareness threshold, *n* is the number of seed patterns, *r* is the number of unmasked bases in the target genome and *t* (a.k.a. 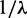) is the scale factor of the score matrix (24). This crudely attempts to make the number of alignments passed to the gapped alignment phase be proportional to the running time of the gapless phase. In our test, *r* = 1 363 595 724 and *t* = 96.1735, so that *d* ranges between 2466 and 1823.

Because LASTZ and LAST are independent implementations with presumably many small differences, it is hard to interpret the different results. As a further comparison, we ran LASTZ with the four weight-11 Iedera seeds (Supplementary Table S1), and *d* = 2000. This performed slightly worse than LAST with the same four patterns (Figure 2I), perhaps because LAST uses adaptive seeds.

### Generality of the results

**Figure 3.**
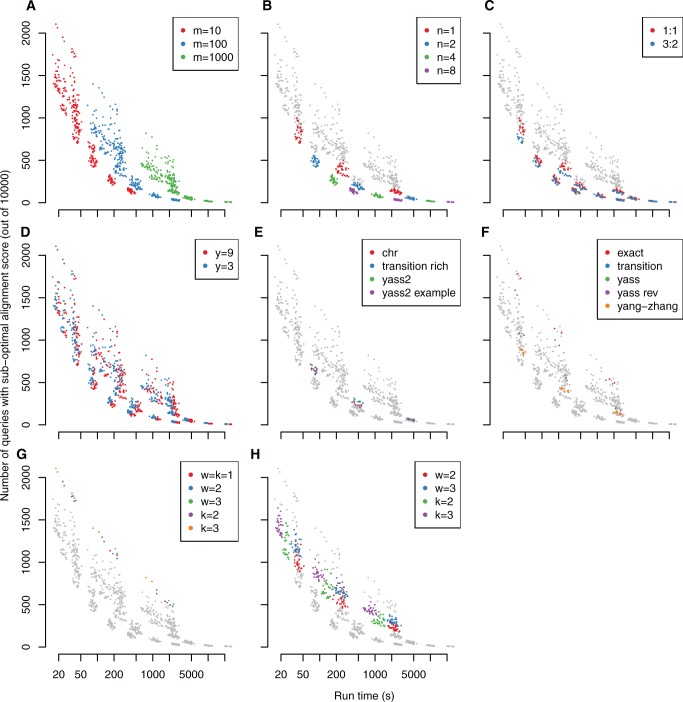
Sensitivity versus speed for aligning human queries to the dog genome with the LAST scoring scheme. Each point represents one combination of algorithmic parameters. Each panel shows the same points, but highlights a different selection of them, according to: rareness threshold m (**A**), number of seeds n (**B**), transition: transversion ratio (**C**), extension threshold y (**D**), predefined seeds (**E** and **F**), and sparse seeding (**G** and **H**).

To check the generality of the above results, we repeated the test using the LAST scoring scheme instead of the LASTZ scheme. The conclusions remain similar (Figure 3), but there are two slight differences. Firstly, the advantage of the 3:2 over the 1:1 patterns is reduced (presumably because the LAST scheme scores transitions the same as transversions), but not eliminated (presumably because there are actually more transitions than transversions). Secondly, it is easier to find these alignments: the optimal alignment scores are missed less often, and the slowest methods give hardly any improvement (i.e. diminishing returns).

We then tried aligning the same human queries to mouse instead of dog (Supplementary Figures S1, S2). The conclusions remain similar. Interestingly, there is no clear sign of diminishing returns with the LASTZ scheme, which suggests that even our most sensitive methods miss many optimal alignments.

We also tried aligning 10 000 random 1-kb chunks of the *melanogaster* genome to the *pseudoobscura* genome (Supplementary Figures S3, S4). In this case, the 1:1 seeds perform better than the 3:2 seeds, as expected. In addition, the seed patterns from YASS are much more competitive because they are suited to the lower transition:transversion ratio. Finally, the exact and all-transition seeds exchange places for worst and second-worst.

### Human–mouse genome comparison

To see how much difference our optimized heuristics make in practice, we compared the whole human and mouse genomes (see ‘Materials and Methods’ section). The resulting alignments include 27 013 whose human sequence has zero overlap with the UCSC human–mouse alignments. This number is a bit misleading because it includes alignments of the same human region to different parts of mouse. If we then merge alignments whose human regions overlap, we end up with 19 019 human segments that are unaligned in UCSC. Some of our new alignments may be paralogs, which are typically not wanted, but because these human regions are otherwise unaligned, it is plausible that many of them are orthologs.

## DISCUSSION

This study’s contributions are practical rather than theoretical. Although we mainly just tested known techniques in a systematic way, we were able to significantly improve large-scale DNA similarity search. The main reason seems to be that previous studies did not thoroughly explore transition seeds. In fact, there are rather few publications on transition seeds, despite their obvious advantage for DNA comparison, and even fewer that consider multiple transition seeds. All these previous studies seem to have considered only seeds with a fixed, low number of transition positions, whereas we show that it is often best to use many transition positions.

One potential shortcoming is that we, of necessity, used Iedera in a heuristic mode that does not guarantee to find optimal seeds for the specified model. The resulting patterns are empirically effective, but we do not know how much more effective they might be with better design, especially for multiple seeds.

It seems best to use as many codesigned patterns as possible, but pushing this further has several difficulties. More patterns need more memory, but terabyte memories exist nowadays, which would allow >100 simultaneous patterns. Perhaps a bigger problem is the difficulty of codesigning so many patterns. Iedera has options to trade run time against how thoroughly it explores the space of patterns, but it is unclear how many patterns it can codesign reasonably well in a feasible time. Finally, our alignment test results exclude the run time needed to preprocess the target genome, which increases linearly with the number of patterns.

We obtained some informative negative results: sparse seeding does not improve performance, and varying the gapless score drop threshold (*y*) has little effect. On the other hand, the gapless score threshold (*d*) is surprisingly important: insertions and deletions are frequent enough that there is often no high-scoring gapless alignment.

An intriguing idea is to use multiple codesigned sparse seeds. For example, we might use two seed patterns at odd-numbered genome positions, and another two at even-numbered positions. This would use the same memory as two nonsparse seeds, but it might improve alignment. (It cannot be any worse because it includes two nonsparse seeds as a special case.) However, some preliminary Iedera results (not shown) suggest that (in the case of spaced seeds) the sensitivity gain obtained is small for this computationally intensive design problem.

Memory can still be a problem (because not everyone has terabyte memories yet, and we may wish to search huge DNA databases), so low-memory indexes (e.g. compressed suffix arrays) are of interest. There has been much research into such indexes (25), but unfortunately little that relates to spaced or transition seeds (9). An interesting possibility is that it might be easier to compress a multiple-seed index than a single-seed index. Another interesting idea for saving memory is ‘neighbor seeds’ (26,27).

Our results also suggest that comparing distant genomes is a hard and unsolved problem. In particular, the human–mouse alignments with the LASTZ scoring scheme (Supplementary Figure S1) do not seem to approach perfect sensitivity even at the longest run times. On the other hand, the easier human–dog and *melanogaster*–*pseudoobscura* alignments do seem to approach perfection (though we cannot be certain because we do not know the guaranteed-maximum alignment scores). The longest run times in these tests extrapolate to ∼12 days for aligning two mammal genomes (if the query chromosomes are aligned in parallel).

We did not consider ‘interpolation’, which is an option in LASTZ to look harder for alignments between previously found alignments. This boosts sensitivity because genomic similarities are often colinear (12). However, some similarities are noncolinear (often the most interesting ones); for many genomes we have only incomplete ‘draft’ sequences with poor contiguity, and in any case interpolation is orthogonal to optimized seeding, so their benefits can be combined.

We hope these results will help to elucidate the evolutionary story of DNA sequences, and also spur other researchers to further improve DNA similarity search, which is still not fully solved.

## SUPPLEMENTARY DATA

Supplementary Data are available at NAR Online.

## FUNDING

Funding for open access charge: AIST (National Institute for Advanced Industrial Science and Technology).

*Conflict of interest statement*. None declared.

## Supplementary Material

Supplementary Data

## References

[1] Brejova B, Brown DG, Vinar T (2004). Optimal spaced seeds for homologous coding regions. J. Bioinform. Comput. Biol..

[2] Zhou L, Stanton J, Florea L (2008). Universal seeds for cDNA-to-genome comparison. BMC Bioinformatics.

[3] Altschul SF, Gish W, Miller W, Myers EW, Lipman DJ (1990). Basic local alignment search tool. J. Mol. Biol..

[4] Altschul SF, Madden TL, Schaffer AA, Zhang J, Zhang Z, Miller W, Lipman DJ (1997). Gapped BLAST and PSI-BLAST: a new generation of protein database search programs. Nucleic Acids Res..

[5] Ma B, Tromp J, Li M (2002). PatternHunter: faster and more sensitive homology search. Bioinformatics.

[6] Zhang L (2007). Superiority of spaced seeds for homology search. IEEE/ACM Trans. Comput. Biol. Bioinform..

[7] Chao KM, Zhang L (2008). Sequence comparison: theory and methods. Computational Biology.

[8] Noe L, Kucherov G (2004). Improved hit criteria for DNA local alignment. BMC Bioinformatics.

[9] Kielbasa SM, Wan R, Sato K, Horton P, Frith MC (2011). Adaptive seeds tame genomic sequence comparison. Genome Res..

[10] Kent WJ (2002). BLAT–the BLAST-like alignment tool. Genome Res..

[11] Morgulis A, Coulouris G, Raytselis Y, Madden TL, Agarwala R, Schaffer AA (2008). Database indexing for production MegaBLAST searches. Bioinformatics.

[12] Schwartz S, Kent WJ, Smit A, Zhang Z, Baertsch R, Hardison RC, Haussler D, Miller W (2003). Human-mouse alignments with BLASTZ. Genome Res..

[13] Ma B, Yao H (2009). Seed optimization for i.i.d. similarities is no easier than optimal Golomb ruler design (earlier version in APBC 2008). Inf. Proc. Lett..

[14] Kucherov G, Noe L, Roytberg M (2006). A unifying framework for seed sensitivity and its application to subset seeds. J. Bioinform. Comput. Biol..

[15] Meyer LR, Zweig AS, Hinrichs AS, Karolchik D, Kuhn RM, Wong M, Sloan CA, Rosenbloom KR, Roe G, Rhead B (2013). The UCSC Genome Browser database: extensions and updates 2013. Nucleic Acids Res..

[16] Chiaromonte F, Yap VB, Miller W (2002). Scoring pairwise genomic sequence alignments. Pac. Symp. Biocomput..

[17] Sheetlin S, Park Y, Spouge JL (2005). The Gumbel pre-factor k for gapped local alignment can be estimated from simulations of global alignment. Nucleic Acids Res..

[18] Frith MC, Hamada M, Horton P (2010). Parameters for accurate genome alignment. BMC Bioinformatics.

[19] Harris RS (2007). Improved Pairwise Alignment of Genomic DNA.

[20] Frith MC (2011). A new repeat-masking method enables specific detection of homologous sequences. Nucleic Acids Res..

[21] Field LM, Lyko F, Mandrioli M, Prantera G (2004). DNA methylation in insects. Insect Mol. Biol..

[22] Sun Y, Buhler J (2006). Choosing the best heuristic for seeded alignment of DNA sequences. BMC Bioinformatics.

[23] Yang J, Zhang L (2008). Run probabilities of seed-like patterns and identifying good transition seeds. J. Comput. Biol..

[24] Yu YK, Altschul SF (2005). The construction of amino acid substitution matrices for the comparison of proteins with non-standard compositions. Bioinformatics.

[25] Vyverman M, De Baets B, Fack V, Dawyndt P (2012). Prospects and limitations of full-text index structures in genome analysis. Nucleic Acids Res..

[26] Csürös M, Ma B (2007). Rapid homology search with neighbor seeds. Algorithmica.

[27] Ilie L, Ilie S (2009). Fast computation of neighbor seeds. Bioinformatics.

