# Supplementary material for: Improved search heuristics find 20 000 new alignments between human and mouse genomes
Source: Nucleic Acids Res. 2014 Jan 31;42(7):e59. doi: 10.1093/nar/gku104 (PMC3985675; doi:10.1093/nar/gku104)
Supplement: Supplementary Data [file supp_42_7_e59__index.html]

Improved search heuristics find 20 000 new alignments between human and mouse genomes — Improved search heuristics find 20 000 new alignments between human and mouse genomes — Supplementary Data 

# Improved search heuristics find 20 000 new alignments between human and mouse genomes

## Supplementary Data

files

**Files in this Data Supplement:**

- Supplementary Data - pdf file
