# Supplementary material for: Improved search heuristics find 20 000 new alignments between human and mouse genomes
Source: Nucleic Acids Res. 2014 Jan 31;42(7):e59. doi: 10.1093/nar/gku104 (PMC3985675; doi:10.1093/nar/gku104)
Supplement: Supplementary Data [file supp_gku104_nar-03355-met-n-2013-File009.pdf]

Supplement to: Improved search heuristics find 20,000 new alignments between human and mouse genomes

Martin C. Frith and Laurent Noé

November 12, 2013

1 Figures

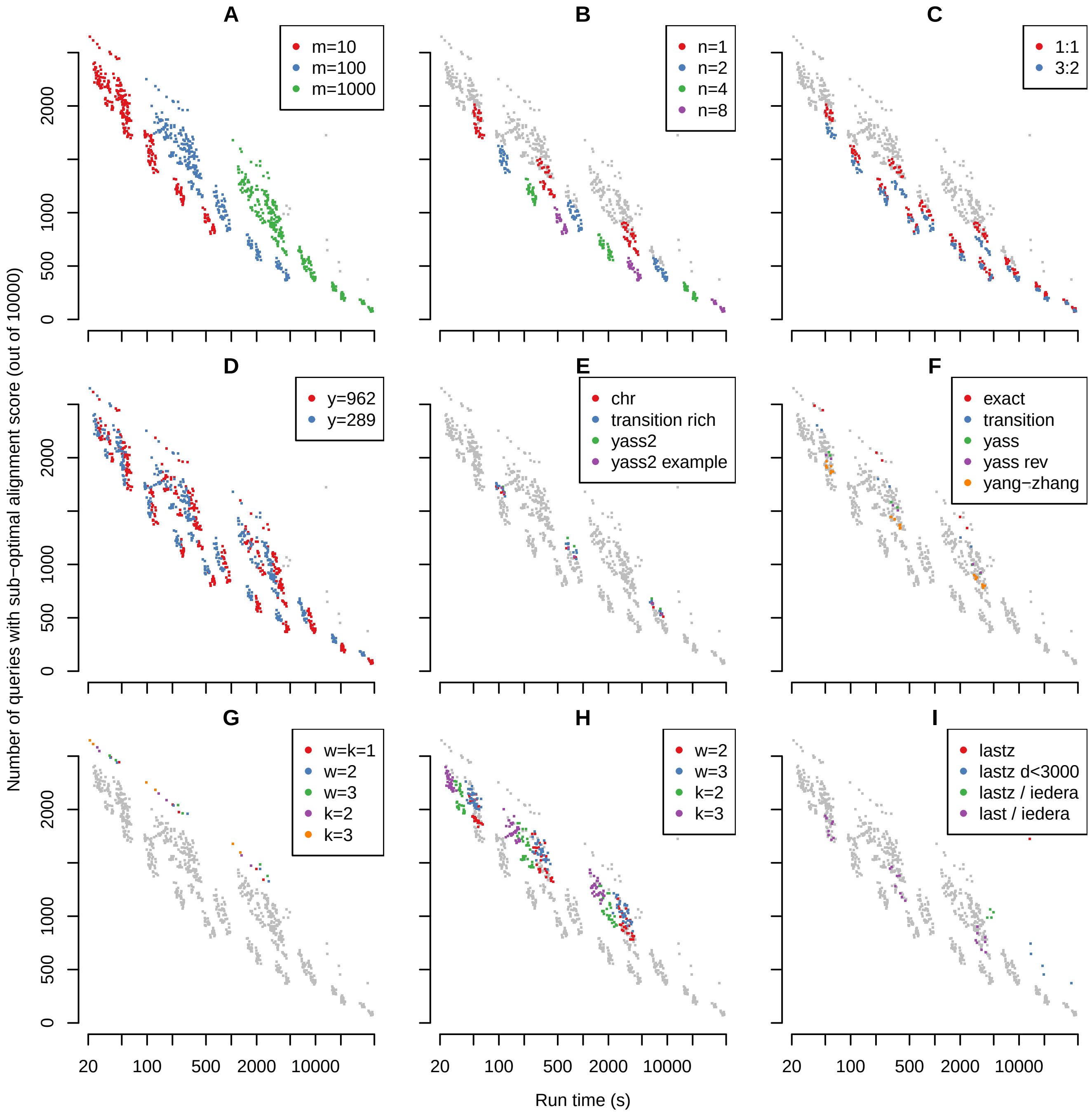

Figure 1: Sensitivity versus speed for aligning human queries to the mouse genome with the LASTZ scoring scheme. Each point represents one combination of algorithmic parameters.

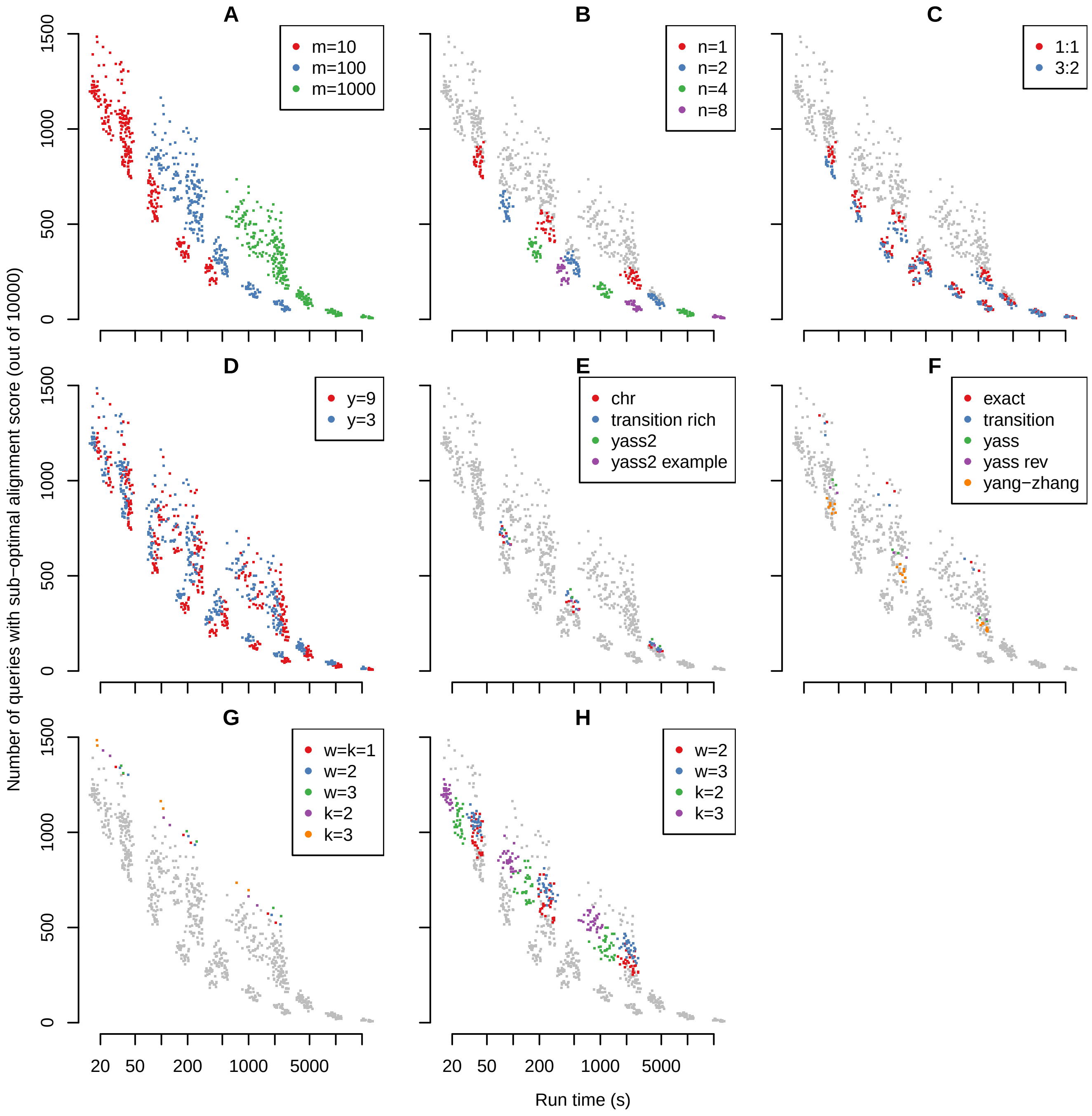

Figure 2: Sensitivity versus speed for aligning human queries to the mouse genome with the LAST scoring scheme. Each point represents one combination of algorithmic parameters.

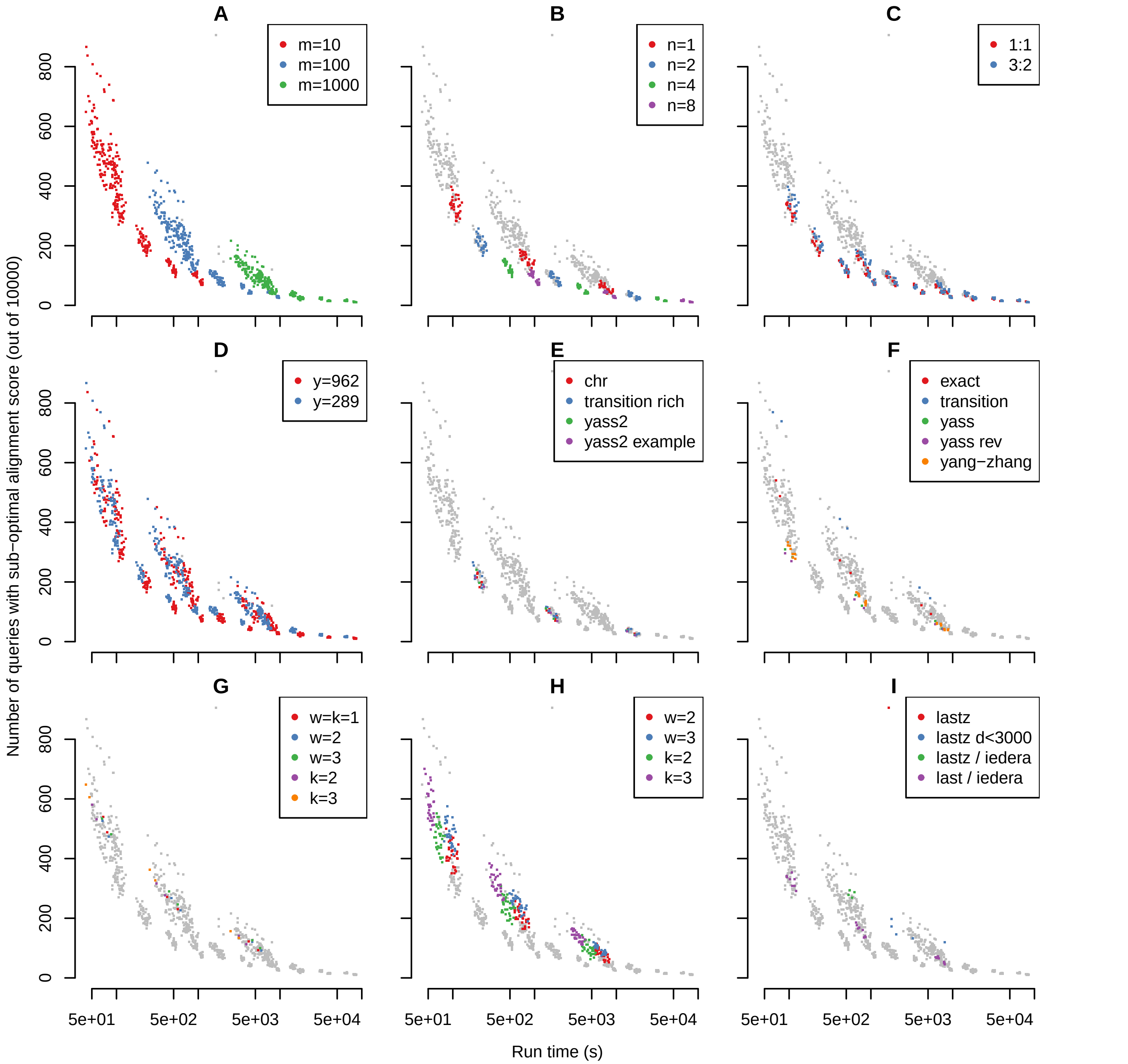

Figure 3: Sensitivity versus speed for aligning *melanogaster* queries to the *pseudoobscura* genome with the LASTZ scoring scheme. Each point represents one combination of algorithmic parameters.

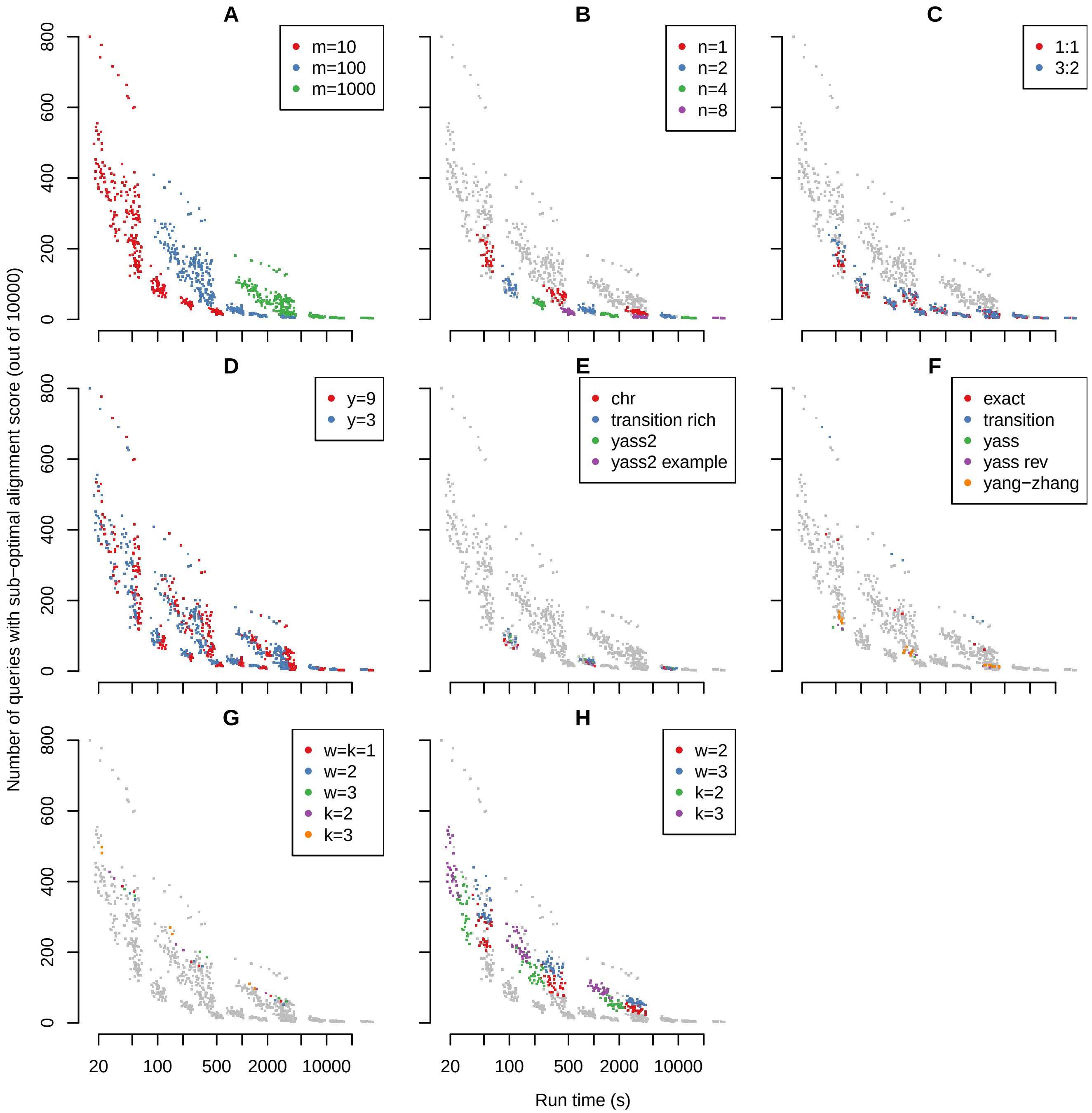

Figure 4: Sensitivity versus speed for aligning *melanogaster* queries to the *pseudoobscura* genome with the LAST scoring scheme. Each point represents one combination of algorithmic parameters.

2 Seed patterns used in this study

2.1 Original seed patterns

Table 1: Single seed patterns designed by Iedera.

transitions: transversions	weight	length	pattern
1:1	9	40	1110T101T0111
1:1	9	64	11T01T001010T1T1
1:1	10	40	111T0T10110111
1:1	10	64	111T001T010T101T1
1:1	11	40	11T101T10T10T111
1:1	11	64	11T1010T1001T0T111
1:1	12	40	111T01T10110T1T11
1:1	12	64	111T01T010T1T001T1T1
3:2	9	40	11TTT0T10T01TTT1
3:2	9	64	11TTT0T0TT01TT0TT1
3:2	10	40	1TTT1T0T01TT0T1TT1
3:2	10	64	1T1T0TTT001T0TT0T1T1
3:2	11	40	11TT0TT10TTT01TT1T1
3:2	11	64	1TT1T0T0TT100TTT0T1TT1
3:2	12	40	1TT1TT10TTT0T1T0TT1T1
3:2	12	64	1TTTT010TT1T00T10TTTTT1T

Table 2: Pairs of seed patterns designed by Iedera.

transitions: transversions	weight	length	patterns
1:1	9	40	1101T01T10111,111TT0T0010010T11
1:1	9	64	1110101T0110T1,1TT10T0T001T00T111
1:1	10	40	1110101101111,11TT1001TT0T01T11
1:1	10	64	111101T10T1011,11T010TT0010T001TT11
1:1	11	40	11101T011T10111,11TTT10T0T0100T01TT11
1:1	11	64	111T01001T00T0TTT0111,11110TT101011011
1:1	12	40	111T10TT0TTT0T10T111,111101110101111
1:1	12	64	1T11T0T1101011T11,111T010100T10000T0T1TT11
3:2	9	40	11TTT0T0TT0T01TTT1,1T10T1T01T111
3:2	9	64	11TTT00TT0T010T1T1,11T011010TT1T1
3:2	10	40	11T1T10110TT11,1T1T0TT0T0TTT0TTTT11
3:2	10	64	1TT1T10TT10T111,11TTT001T0T0TT01TTT1
3:2	11	40	1TTT1TT00T010TTT0TTT11,1T110T1TT01T1T11
3:2	11	64	T1TT01T00TTTT0T01T0111,1TT10TTT10010TT10TTTTT
3:2	12	40	11TTT10T0T0TT0T00TT1TT11,11TTTT1T01T101TT11
3:2	12	64	11TT1T0T1T10TT1T11,11TTT1T00TT0100TT0TTT1T1

Table 3: Sets of 4 seed patterns designed by Iedera.

transitions: transversions	weight	length	patterns
1:1	9	40	111T01001010T11,1T1T1011TT11,1101001100T01T11,11T0T1000T100T0111
1:1	9	64	1101T0101TTT11,111T0T00TTT00T1011,11T001T10001T01T1,1110110010111
1:1	10	40	111T000T010001011TT1,1T1T11T0T11011,11T0101000T1TT0111,1110TT011010T1T1
1:1	10	64	1TTT10001T0T001101T1,11T1TT1T0T10T11,1110100101001T1T1,1110010T00T10T0T0111
1:1	11	40	1111T000TT0T00T101T11,1TTTT01T010001T0T0T111,11T10110011T111,111011TTT01010T11
1:1	11	64	1111001000100100TT111,11101T1011T111,111T01T0101100111,11TTT0T10T00TTT0101TT1
1:1	12	40	111010T101T0010T011T1,11T110001001T1T1T11,111TTT0T0TT00T010001T111,1T10T11T1T0110111
1:1	12	64	11TTTT0T100T0T0T10TT0111,1TT10T011101101T11,110111T00TT101T111,11T11010001001T000T1TTT1
3:2	9	40	1110T0TTTT0TT0TT1T,1TTTT01001T0010T11,1T110111T11,11T10T00T101T11
3:2	9	64	1T1TT1T0T1T01TT,TTTT100100T1010T11,1100TT0101T0T0T1T1,111T0010T000T101T1
3:2	10	40	11TT0TTT0TTT010TT11,1T101T00T1000T1TTTT1,11T110T101111,11TT10101T0TTT11
3:2	10	64	11100TT01T00T10TTTT1,TTTT110TT0T001T0T1T1,11TT010T01TT0001T11,11TT10T1T101TT1
3:2	11	40	1TTT0T11T01TT01T11,1TTT100T0TTT0TT0T10111,1111T0TT0010T0010TTTT1,11T1010T1T0T11TT1
3:2	11	64	T1T10T1100T1TTT1T1,1T01101T00T0T1T0T0TT11,1TT101TTT0TT00T010111,11TTTT0010T01T010TTTT1
3:2	12	40	11TTTTT0T1T01010T111,1TT1TTT00T1T00T0TTT01T11,111T1101T0T1TTT11,111T0T101000TT0T001TTT11
3:2	12	64	TT1TT1000T1T00T01010T111,11TTT0TT10T0T0TT0T10TT11,11TT10110TT1T1T11,1T1011TT0T001T01TT0TTTTT

Table 4: Sets of 8 seed patterns designed by Iedera.

transitions: transversions	weight	length	patterns
1:1	9	40	1T1101T0TTT1T1,111010T0TT0T0111,1TT1TT00101010011,1TTT0011T00T110T1<br>1110T100001T0TTTT1,11T111T111,110T0T01T0010011T1,1T100T10T100T01011
1:1	9	64	111010100011011,TT1TTT0TT0101T11,11101101TT11,111001T0T0T0TTT011<br>11TT1TT1111,1TT10T01001T00T1T1,11100010010T1001T1,111T111T11
1:1	10	40	111T100T001TTT0T11,1110T1010TT011T1,110101T1100T111,11011TT0111011<br>1T11T01T11T101,1110T11011T11,1110100T100000101T11,11T011T0T0100011T1
1:1	10	64	111T0TT01001011T1,1T0110T10001100111,1110TT11T1T11,11100T10T0T0TT0TTT11<br>1TTT0111010TT1T1,1T1T110110111,111010010TT10T1T1,11TTT000101T00100111
1:1	11	40	11T100101001TT001TTT1,110T10T0T010T0TT001111,111T100T1T1T0111,11TTTTTT10T010T111<br>1TTTT1000T1001001TTT11,110111101T1T11,1111001T00T0010101011,1T10T1T01T0T00110TTT1
1:1	11	64	11T10TTTT00TT1010T11,1T1TT0T01T1100T1011,1TT1TT0T1T0T1T1TT1,11TT0101000010T01T0111<br>11TT110100110111,11T10T001TTT0010TTT11,111011001010TT0T11T,1101T1T111TT11
1:1	12	40	1T111T01001101111,1101101TT1T1T10TT1,11T1011T0T001010011T1,1110TT0T10T01TTT01T11<br>11T01000111000101TTT11,11T1T0T1100TT00T0T10T11,11TT01T1T1T00T1111,11TT10100T0T0TTT0T01TT11
1:1	12	64	1TTT10TT010T1TT00T0TTT11,11T10TT00T011TT01T1T1,1101T1010100TT0101T11,111T11011T1111<br>1TTT0T1100100T110TTT011,1110TTT001T0T00TT0110T11,1110T011T0010100T1T0TT1,1T1100TTTTTT0011T01011
3:2	9	40	11T0T10TTT011T1,1T1T0T001100011T1,11101001T00T00TT11,1TT101101111<br>1T1110TT1TTT1,11T01TT010TT0T11,110TTTT00T01T10TT1,1TTT010100T01T0T11
3:2	9	64	1T111000T001T0T1TT,11T0T00T101010TTT1,110T101TT00T0T0T11,110T0T1001T0T01TT1<br>1T10110101TTT1,TT100110T0TT100T11,1TTTTT1TTT1011,T1TT00T01T0001111T
3:2	10	40	1T110100T100010111,1110T10110T111,11TTT00TT10TT0TT01T1,1TT1TT010TTT1011T<br>1T0111T0T11T11,1TT0T1T0T0T0TT1T0T11,111001T00T01100T1T1,1T10101T00100101TT1
3:2	10	64	110010T101T001TT0TT1,1TTTT100TTTTT0TT01T1,1T101T100TT101011,1TTT11TT1010T11<br>1110T00T11T01T0T1T,T110T1TT0T00TTT1TT1,11TT0TTT000101001T11,1T11000100T10T1T011
3:2	11	40	11T10T100T1TTT0TT11,11T11T10101111,11T0T10T0100TTT1TT1TT,1110TT0110110111<br>1T1T1011T0TT10T11,1TTT1T0T0T0T001TT0T111,11T100TTT00TT0T0101T11,1T1TTT01TTT001001TTT1
3:2	11	64	11T10T10T0010TTT0TTTT1,1TTTT0T1001TT00TT101T1,T1110TT1T010T01T11,11T01010T0T01001TTT11<br>111T100001T00T100T1011,11T0110T1T0TT01T11,1T1TTTTT0T00110T0111,1T1T011T1011T11
3:2	12	40	1101T1T0T1T00TT1TT11,1TTTTT010TT0TT01011TTT1,1TTTT10010T011T0TTTT11,111T011T0T01T100111<br>1T10T100TT01000TT01TT111,111T101TT000T0T10T00T1T1,111100T011TTT00T0TT01T1,1T1T10T11011011T1
3:2	12	64	11T10100TT00TT01010T1TT1,1TT10011T0T010T01T0111,1110T0T00TTT100T0TT11011,11TTTTT101T000T1T0TTTT1<br>1T10110100T1TT0T0T1TT1,11TT0T101T011001111,111011T0T101T11T1,T1TT1T0TT0T01010T100TT11

Table 5: Seed patterns designed by Iedera for 1:2 sparsity.

transitions: transversions	weight	length	pattern
1:1	9	40	11110011TT11
1:1	9	64	11TTTT001100TT11
1:1	10	40	11110TT011TT11
1:1	10	64	11T1001T00T11T11
1:1	11	40	11110011TT1111
1:1	11	64	111TT10011TTTT11
1:1	12	40	111TT10011TT1111
1:1	12	64	111TTT011001TT1T11
3:2	9	40	11TTTTTT00TTTT11
3:2	9	64	1TTT01T0TT00TTT11T
3:2	10	40	11TTTTT00TTTTTTT11
3:2	10	64	11TTTT0TT0TT00TTTT11
3:2	11	40	11TTTTTT00TTTTTTTT11
3:2	11	64	1TTTT10TT0TT0TT0T1TT1T
3:2	12	40	11TTTTTT0011TTTTTT11
3:2	12	64	11TTTTTT00TTTT0TT0TTTT11

Table 6: Seed patterns designed by Iedera for 1:3 sparsity.

transitions: transversions	weight	length	pattern
1:1	9	40	111TTT01TTT11
1:1	9	64	1110TTTTTT111
1:1	10	40	111TTT1TTT111
1:1	10	64	111TTT10T0T0T111
1:1	11	40	111T111TTT111
1:1	11	64	111TTT1100TTT111
1:1	12	40	111TT111T0TTT111
1:1	12	64	111TT111T0TTT111
3:2	9	40	1T1TTT0T0T1TTTT1
3:2	9	64	T1TTTT000TTT1TTTT1
3:2	10	40	T1TTTTTTT0011TTTTTT
3:2	10	64	111TTT000TTTTTTTTT1
3:2	11	40	111TTTTTTTTTT111
3:2	11	64	1T1TTTTTT0T0T0T0TTT1T1
3:2	12	40	111TTTTTT0TTTTTT111
3:2	12	64	TT1T1T0TTTTT1000TTTTTTT1

Table 7: Pairs of seed patterns suggested previously.

name	patterns
chr-avg	110101T10100T111,1011T001000010011T11
chr-best	10111T1001010T11,111T0101000000T1010011
chr-inver-fungi	1T0110110100T0111,1011T0001100010001T101
transition rich large	1T1001000011100T011,10101T0001010T011011
transition rich small	11T101011T0111,1011001001T100T0111
transition rich vsmall	111T1101101T1,1110T0100T0011111
transition rich	1110100001010001T10T1,110100T101011100T1
yass2	11101T011T11,111001010010111
yass2 example	110101T1011T011,11T100T00110100111

Table 8: Single seed patterns suggested previously.

name	pattern
yass	1T10011001011T1
yass rev	1T11010011001T1
yang-zhang B4.4.09	1110T0T1001T01T1
yang-zhang B4.4.10	111T001T010T101T1
yang-zhang B4.4.11	111T01010T1001TT11
yang-zhang B4.4.12	111TT011001T0101T11

2.2 Trimmed seed patterns

The following patterns were obtained from the preceding ones by taking the shortest prefix that reconstructs the original pattern upon cyclic extension.

Table 9: Single seed patterns designed by Iedera.

transitions: transversions	weight	length	pattern
1:1	9	40	1110T101T0
1:1	9	64	11T01T001010T1T
1:1	10	40	111T0T10110
1:1	10	64	111T001T010T101T
1:1	11	40	11T101T10T10T1
1:1	11	64	11T1010T1001T0T1
1:1	12	40	111T01T10110T1T
1:1	12	64	111T01T010T1T001T1T
3:2	9	40	11TTT0T10T01TTT
3:2	9	64	11TTT0T0TT01TT0TT
3:2	10	40	1TTT1T0T01TT0T1TT
3:2	10	64	1T1T0TTT001T0TT0T
3:2	11	40	11TT0TT10TTT01TT1T
3:2	11	64	1TT1T0T0TT100TTT0T
3:2	12	40	1TT1TT10TTT0T1T0TT1T
3:2	12	64	1TTTT010TT1T00T10TTTTT

Table 10: Pairs of seed patterns designed by Iedera.

transitions: transversions	weight	length	patterns
1:1	9	40	1101T01T101,111TT0T0010010T
1:1	9	64	1110101T0110T,1TT10T0T001T00T11
1:1	10	40	1110101101,11TT1001TT0T01T
1:1	10	64	111101T10T10,11T010TT0010T001TT
1:1	11	40	11101T011T10,11TTT10T0T0100T01TT
1:1	11	64	111T01001T00T0TTT0,11110TT1010110
1:1	12	40	111T10TT0TTT0T10T,11110111010
1:1	12	64	1T11T0T110101,111T010100T10000T0T1TT
3:2	9	40	11TTT0T0TT0T01TTT,1T10T1T01T11
3:2	9	64	11TTT00TT0T010T1T,11T011010TT1T
3:2	10	40	11T1T10110TT,1T1T0TT0T0TTT0TTTT1
3:2	10	64	1TT1T10TT10T11,11TTT001T0T0TT01TTT
3:2	11	40	1TTT1TT00T010TTT0TTT1,1T110T1TT01T
3:2	11	64	T1TT01T00TTTT0T01T0111,1TT10TTT10010TT10TTTTT
3:2	12	40	11TTT10T0T0TT0T00TT1TT,11TTTT1T01T101TT
3:2	12	64	11TT1T0T1T10TT1T,11TTT1T00TT0100TT0TTT1T

Table 11: Sets of 4 seed patterns designed by Iedera.

transitions: transversions	weight	length	patterns
1:1	9	40	111T01001010T,1T1T1011TT1,1101001100T01T,11T0T1000T100T01
1:1	9	64	1101T0101TTT,111T0T00TTT00T10,11T001T10001T01T,1110110010
1:1	10	40	111T000T010001011TT,1T1T11T0T1101,11T0101000T1TT01,1110TT011010T1T
1:1	10	64	1TTT10001T0T001101T,11T1TT1T0T10T,1110100101001T1T,1110010T00T10T0T0
1:1	11	40	1111T000TT0T00T101T,1TTTT01T010001T0T0T11,11T10110011T1,111011TTT01010T
1:1	11	64	1111001000100100TT,11101T1011T,111T01T0101100,11TTT0T10T00TTT0101TT
1:1	12	40	111010T101T0010T011T,11T110001001T1T1T,111TTT0T0TT00T010001T,1T10T11T1T011011
1:1	12	64	11TTTT0T100T0T0T10TT01,1TT10T01110110 1T1,110111T00TT101T1,11T11010001001T000T1TTT
3:2	9	40	1110T0TTTT0TT0TT1T,1TTTT01001T0010T1,1T11011,11T10T00T101T
3:2	9	64	1T1TT1T0T1T01TT,TTTT100100T1010T11,1100TT0101T0T0T1T,111T0010T000T101T
3:2	10	40	11TT0TTT0TTT010TT,1T101T00T1000T1TTTT,11T110T1011,11TT10101T0TTT
3:2	10	64	11100TT01T00T10TTTT,TTTT110TT0T001T0T1T1,11TT010T01TT0001T,11TT10T1T101TT
3:2	11	40	1TTT0T11T01TT01T1,1TTT100T0TTT0TT0T1011,1111T0TT0010T0010TTTT,11T1010T1T0T11TT
3:2	11	64	T1T10T1100T1TT,1T01101T00T0T1T0T0TT1,1TT101TTT0TT00T01011,11TTTT0010T01T010TTTT
3:2	12	40	11TTTTT0T1T01010T1,1TT1TTT00T1T00T0TTT01T1,111T1101T0T1TTT,111T0T101000TT0T001TTT
3:2	12	64	TT1TT1000T1T00T01010T111,11TTT0TT10T0T0TT0T10TT,11TT10110TT1T1T,1T1011TT0T001T01TT0TTTTT

Table 12: Sets of 8 seed patterns designed by Iedera.

transitions: transversions	weight	length	patterns
1:1	9	40	1T1101T0TTT,111010T0TT0T0,1TT1TT0010101001,1TTT0011T00T110T<br>1110T100001T0TTTT,11T1,110T0T01T0010011T,1T100T10T100T0101
1:1	9	64	1110101000110,TT1TTT0TT0101T11,11101101TT,111001T0T0T0TTT0<br>11TT1TT11,1TT10T01001T00T1T,11100010010T1001T,111T
1:1	10	40	111T100T001TTT0T,1110T1010TT011T,110101T1100T1,11011TT01<br>1T11T01T11T10,1110T11011T,1110100T100000101T,11T011T0T0100011T
1:1	10	64	111T0TT01001011T,1T0110T1000110011,1110TT11T1T,11100T10T0T0TT0TTT<br>1TTT0111010TT1T,1T1T11011011,111010010TT10T1T,11TTT000101T001001
1:1	11	40	11T100101001TT001TTT,110T10T0T010T0TT0011,111T100T1T1T0,11TTTTTT10T010T1<br>1TTTT1000T1001001TTT1,110111101T1T,1111001T00T00101010,1T10T1T01T0T00110TTT
1:1	11	64	11T10TTTT00TT1010T,1T1TT0T01T1100T101,1TT1TT0T1T0T1T,11TT0101000010T01T01<br>11TT1101001101,11T10T001TTT0010TTT,111011001010TT0T11T,1101T1T111TT
1:1	12	40	1T111T0100110111,1101101TT1T1T10TT,11T1011T0T0010100,1110TT0T10T01TTT01T<br>11T01000111000101TTT,11T1T0T1100TT00T0T10T,11TT01T1T1T00T11,11TT10100T0T0TTT0T01TT
1:1	12	64	1TTT10TT010T1TT00T0TTT1,11T10TT00T011TT01T1T,1101T1010100TT0101T,111T11011T1<br>1TTT0T1100100T110TTT01,1110TTT001T0T00TT0110T,1110T011T0010100T1T0TT,1T1100TTTTTT0011T0101
3:2	9	40	11T0T10TTT011T,1T1T0T00110001,11101001T00T00TT,1TT10110111<br>1T1110TT1TTT,11T01TT010TT0T,110TTTT00T01T10TT,1TTT010100T01T0T1
3:2	9	64	1T111000T001T0T1TT,11T0T00T101010TTT,110T101TT00T0T0T,110T0T1001T0T01TT<br>1T10110101TTT,TT100110T0TT100T11,1TTTTT1TTT101,T1TT00T01T0001111
3:2	10	40	1T110100T10001011,1110T10110T,11TTT00TT10TT0TT01T,1TT1TT010TTT101<br>1T0111T0T11T1,1TT0T1T0T0T0TT1T0T1,111001T00T01100T1T,1T10101T00100101TT
3:2	10	64	110010T101T001TT0TT,1TTTT100TTTTT0TT01T,1T101T100TT10101,1TTT11TT1010T1<br>1110T00T11T01T0T1T,T110T1TT0T00TTT1T,11TT0TTT000101001T,1T11000100T10T1T01
3:2	11	40	11T10T100T1TTT0TT,11T11T101011,11T0T10T0100TTT1TT1TT,1110TT0110110<br>1T1T1011T0TT10T1,1TTT1T0T0T0T001TT0T11,11T100TTT00TT0T0101T,1T1TTT01TTT001001TTT
3:2	11	64	11T10T10T0010TTT0TTTT,1TTTT0T1001TT00TT101T,T1110TT1T010T01,11T01010T0T01001TTT<br>111T100001T00T100T10,11T0110T1T0TT01T,1T1TTTTT0T00110T011,1T1T011T1011T1
3:2	12	40	1101T1T0T1T00TT1TT,1TTTTT010TT0TT01011TTT,1TTTT10010T011T0TTTT1,111T011T0T01T100<br>1T10T100TT01000TT01TT11,111T101TT000T0T10T00T1T,111100T011TTT00T0TT01T,1T1T10T1101101
3:2	12	64	11T10100TT00TT01010T1TT,1TT10011T0T010T01T011,1110T0T00TTT100T0TT110,11TTTTT101T000T1T0TTTT<br>1T10110100T1TT0T0T1TT,11TT0T101T0110011,111011T0T101T11T,T1TT1T0TT0T01010T100TT11

Table 13: Seed patterns designed by Iedera for 1:2 sparsity.

transitions: transversions	weight	length	pattern
1:1	9	40	11110011TT
1:1	9	64	11TTTT001100TT
1:1	10	40	11110TT011TT
1:1	10	64	11T1001T00T11T
1:1	11	40	11110011TT
1:1	11	64	111TT10011TTTT
1:1	12	40	111TT10011TT1
1:1	12	64	111TTT011001TT1T
3:2	9	40	11TTTTTT00TTTT
3:2	9	64	1TTT01T0TT00TTT1
3:2	10	40	11TTTTT00TTTTTTT
3:2	10	64	11TTTT0TT0TT00TTTT
3:2	11	40	11TTTTTT00TTTTTTTT
3:2	11	64	1TTTT10TT0TT0TT0T1TT
3:2	12	40	11TTTTTT0011TTTTTT
3:2	12	64	11TTTTTT00TTTT0TT0TTTT

Table 14: Seed patterns designed by Iedera for 1:3 sparsity.

transitions: transversions	weight	length	pattern
1:1	9	40	`111TTT01TTT`
1:1	9	64	`1110TTTTTT`
1:1	10	40	`111TTT1TTT`
1:1	10	64	`111TTT10T0T0T`
1:1	11	40	`111T111TTT`
1:1	11	64	`111TTT1100TTT`
1:1	12	40	`111TT111T0TTT`
1:1	12	64	`111TT111T0TTT`
3:2	9	40	`1T1TTT0T0T1TTTT`
3:2	9	64	`T1TTTT000TTT1TTT`
3:2	10	40	`T1TTTTTTT0011TTTTT`
3:2	10	64	`111TTT000TTTTTTTTT`
3:2	11	40	`111TTTTTTTTTT`
3:2	11	64	`1T1TTTTTT0T0T0T0TTT`
3:2	12	40	`111TTTTTT0TTTTTT`
3:2	12	64	`TT1T1T0TTTTT1000TTTTT`

Table 15: Pairs of seed patterns suggested previously.

name	patterns
chr-avg	110101T10100T1,1011T001000010011T1
chr-best	10111T1001010T1,111T0101000000T10100
chr-inver-fungi	1T0110110100T011,1011T0001100010001T
transition rich large	1T10010000111 00T01,10101T0001010T01101
transition rich small	11T101011T01,1011001001T100T011
transition rich vsmall	111T1101101T,1110T0100T0011
transition rich	111010000101001T10T,110100T101011100T
yass2	11101T011T,111001010010
yass2 example	110101T1011T0,11T100T001101001

Table 16: Single seed patterns suggested previously.

name	pattern
yass	1T1001100101
yass rev	1T1101001100
yang-zhang B4.4.09	1110T0T1001T01T
yang-zhang B4.4.10	111T001T010T101T
yang-zhang B4.4.11	111T01010T1001TT
yang-zhang B4.4.12	111TT011001T0101T
